# The Interplay of Coping Strategies and Quality of Life in Inflammatory Bowel Disease: A Systematic Review

**DOI:** 10.34172/mejdd.2025.409

**Published:** 2025-01-31

**Authors:** Lining Wang, Norafisyah Makhdzir, Zarina Haron

**Affiliations:** ^1^Department of Nursing, Faculty of Medicine and Health Sciences, Universiti Putra Malaysia, Selangor, Malaysia

**Keywords:** Inflammatory bowel disease, Coping strategies, Quality of life, Coping theory framework

## Abstract

**Background:**

This systematic review, grounded in coping theory, examines the relationship between coping strategies and quality of life (QoL) in patients with inflammatory bowel disease. It synthesizes current findings and guides future research to address existing knowledge gaps.

**Methods:**

A comprehensive search of Scopus, PubMed, Google Scholar, and Web of Science (from April 2000 to April 2024) identified 17 peer-reviewed studies.

**Results:**

Coping strategies directly and indirectly influence the QoL of patients with inflammatory bowel disease (IBD). Adaptive strategies, such as problem-solving and seeking social support, consistently improve QoL even in patients with active disease by promoting better disease management and emotional well-being. Maladaptive strategies, such as avoidance and emotional detachment, lead to decreased QoL and increased psychological distress. In addition, self-efficacy and psychological resilience play a key role in mediating the relationship between coping and QoL. However, coping strategies differ by disease stage and between demographic groups.

**Conclusion:**

This study identifies significant gaps in research, such as limited multi-center and longitudinal studies, cross-cultural inconsistencies, and insufficient focus on specific populations. These gaps restrict understanding of the long-term effects of coping strategies. Future research should combine quantitative and qualitative methods to better capture patients’ experiences, aiding the development of personalized interventions.

## Introduction

 Inflammatory bowel disease (IBD) is a chronic inflammatory condition of the gastrointestinal tract, primarily encompassing ulcerative colitis (UC) and Crohn’s disease (CD), both characterized by recurrent episodes of intestinal inflammation. In the past, IBD was typically associated with persons who were Caucasian, European, and lived in Western developed countries.^1,2^ However, the prevalence of IBD has risen dramatically because of changes in lifestyle and the process of global industrialization. The Global Burden of Disease Report indicates that 6.8 million people worldwide-mostly young and middle-aged adults-struggle with IBD.^3^ IBD remains incurable, with treatment objectives focused on controlling inflammation, maintaining remission, and reducing relapses. The unpredictability of the disease severely impacts patients’ daily activities and quality of life (QoL). Missed work, reduced productivity, and task completion difficulties often result from sudden flare-ups, frequent toileting, fatigue, and pain experienced by patients with IBD.^4^ Therefore, for patients with IBD, QoL has become a key indicator of how well their condition is being managed. Some studies have found that people with IBD who use unhealthy ways to deal with stress are more likely to have a low QoL, including passive coping, low acceptance, emotion-focused coping, negative religious coping, low disease acceptance, perceived lack of disease control, resignation, and so on.^2^

 This literature review aims to explore and clarify the relationship between coping strategies and QoL among patients with IBD within the framework of coping theory. Analyzing existing research seeks to lay the groundwork for healthcare professionals to develop relevant management and intervention strategies. The goal is to inform the design of approaches that can potentially improve the QoL for patients with IBD, addressing their complex needs through targeted and evidence-based measures.

## Materials and Methods

###  Search Strategy

 This literature search was conducted using four English databases, Scopus, PubMed, Google Scholar, and Web of Science, from April 2000 to April 2024. Truncation symbols (e.g., *), Medical Subject Headings (MeSH), and relevant entry terms were employed during the search process to refine keywords aligned with the study’s objectives. Key terms such as “inflammatory bowel disease,” “ulcerative colitis,” “Crohn’s disease,” “quality of life,” “coping,” and “coping strategies” were used. These terms were searched both individually and in combination through Boolean operators (‘AND’ ‘OR,’ and ‘NOT’) to ensure a comprehensive retrieval of relevant literature.

###  Study Selection

 The literature reviewed included original research articles published in peer-reviewed journals. The inclusion criteria were as follows: (1) studies that used standardized tools to assess the coping strategies adopted by patients with IBD; (2) studies that reported and analyzed the associations between coping measures. Exclusion criteria included books or book chapters, duplicate publications, inaccessible full-text articles, case reports, and experimental studies. Databases were manually searched for additional literature, and reference management was performed using EndNote 21 software.

## Results

**Figure 1:**
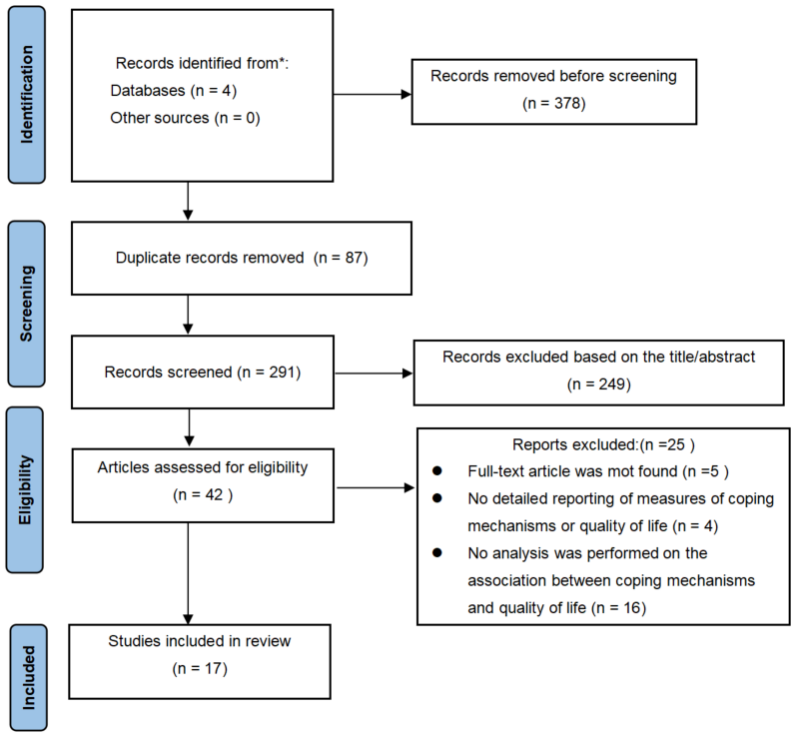
PRISMA flowchart of article identification and selection process

 The study selection process followed the PRISMA framework, as shown in Figure 1. 17 articles were included in this review. Table 1 provides a detailed summary of these studies, including the authors, publication years, participant demographics, study designs, instruments used to assess coping and QoL, and the key findings regarding their relationship.

**Table 1:** Characteristics of included literature

**(Author, Year)**	**Sample** **(male/female)**	**Study Design**	**(Cope/QoL)** **measures**	**Relationship between Cope and QoL**	**Main findings**
Luo, 2018^5^	116/113	Prospective cohort study	MCMQ/ IBDQ	Lower QoL was associated with higher PSS scales and the desirability of an acceptance-resignation response.	The manifestation of acceptance-resignation behavior was associated with a reduced QoL and a poorer prognosis among patients with UC.
Chao, 2019^6^	88/119	Cross-sectional study	Brief- COPE/ SIBDQ	A lower QoL was associated with disease activity and unhealthy ways of dealing.	Adaptive coping mechanisms and Disease Activity were each independently associated with patient poor prognoses. In contrast, patients with higher self-efficacy were positively associated with adaptive coping mechanisms.
Gandhi, 2014^7^	29/41	Cross-sectional study	CISS / IBDQ	Patients with active disease had a much lower QoL than those with inactive disease.	People with IBD who did not have active disease were less likely to use task-oriented coping than people with active disease. There was also a lower chance of return when people used active coping styles.
Jones, 2006^8^	20/28	Cross-sectional study	WOC/ IBDQ	The QoL of patients with active disease was much lower than that of patients with inactive disease. There were no significant differences in HRQOL between patients with IBD or IBS. However, HRQOL was lower in both IBD and IBS patients compared with controls.	People with IBD are far less likely than healthy people to use coping styles such as strategic problem-solving and active reflection.
Kantidakis, 2021^9^	63/198	Cross-sectional study	Brief-COPE/ IBDQ	With increased IBD activity, the patient's perceived QoL decreases.	Psychological variables, including maladaptive coping styles and illness beliefs, statistically influence the associations of IBD symptoms with sadness and anxiety and between IBD symptoms and QoL.
McCombie, 2016^10^	Part A: 44/155 Part B: 15/43 Part C: 82/97	Methodological study	IBD-Cope, Brief- COPE/ IBDQ	Elevated scores in maladaptive coping were associated with reduced HRQOL.	Explicitly designed for IBD patients, the IBD-Cope is a short questionnaire with demonstrated dependability and validity.
Parekh, 2015^11^	77/73	Cross-sectional study	JCS/ SIBDQ	patients with IBD mostly rely on emotionally charged coping mechanisms. While depression is related to escape-avoidance coping, anxiety is connected to emotion-focused coping. Notably, the coping strategy used has small effect on stress levels.	Adaptive coping styles are most widely used among patients with IBD.
Rudnik, 2019.^12^	19/14	Preliminary report	FCSQ-14/ SWLS	Coping with stress and cognitive flexibility improves QoL.	The cognitive flexibility and resilience in coping with stress were positively correlated with QoL.
McCombie, 2015^13^	27/27	Prospective observational study	Brief COPE /SIBDQ, SF-12	At 6 months after diagnosis, a significant improvement in patients' HRQOL was observed.	QoL is lower in individuals who use maladaptive coping strategies, which are linked to increased anxiety, depression, and poorer physical outcomes.
Sarid, 2017^14^	158/244	Cohort study	Brief- COPE/ SWLS, SIBDQ	Men with an active disease have higher QoL scores than women. Economic status, coping barriers, and the number of children all affect QoL.	Women were more likely than men to use problem-focused, emotion-focused, and problematic ways of dealing. SWLS and SIBDQ scores were lower in people whose illness was still ongoing.
Zhang, 2018^15^	45/37	Prospective study	BIPQ, BCOPR/ QoL	QoL significantly improved in IBD patients after treatment.	Maladaptive coping is closely linked to the physical and psychological status of patients, and it shows a significant decrease following effective treatment.
Reed, 2021^16^	76/71	Cross-sectional study	Passive Coping Scale/ IMPACT-III,	Youths with worse HRQOL also reported using passive coping strategies more frequently.	Pediatric patients' general self-esteem impacts their QoL through reactive coping methods, especially unhealthy ways of thinking such as catastrophizing.
Sheehan, 2023^17^	72/86	Cross-sectional study	JCS / QOL, SIBDQ	The number of IBD flare-ups was associated with lower QoL in patients.	The main mechanisms adopted by respondents were confronting, evasive, optimistic, and fatalistic.
van Erp, 2017^18^	84/127	Cross-sectional study	CORS/ SF-36, WPAI	Perceptions of illness and coping mechanisms significantly impacted QoL.	People think that things cause IBD in a person's mind and behavior. A person's mental QoL is directly affected by how well they understand their sickness and how much they feel about it. A person's physical QoL is directly affected by how bad they think the effects are and how active their illness is.
Popa, 2022^19^	26/44	Case-control study	COPE questionnaire/SF-36	Problem- and emotion-focused coping improves the QoL.	The IBD group had more stress and a lower QoL, except for social role performance. Problem- and emotion-focused ways of dealing were seen as better than unhealthy ways of coping and abusing drugs or drinking.
Freitas, 2015^20^	63/84	Cross-sectional study	Brief COPE Scale/ WHOQOL-Brief,	Depression and anxiety are independent predictors of QoL.	Religious struggle was associated with symptoms of psychological distress and lower treatment adherence, whereas active religious coping had a positive impact on health satisfaction.
Haapamäki, 2018^21^	150/206	Cross-sectional study	Mastery Scale/K-10	Not describe	Patients with active IBD are more likely to use poorly adapted coping methods.

QoL: Quality of Life, IBD: Inflammatory Bowel Disease, IBDQ: inflammatory Bowel Disease Questionnaire MCMQ: Medical Coping Modes,Questionnaire, Brief COPE – Brief Coping Orientation to Problems Experienced, SIBDQ: Short Inflammatory Bowel Disease Questionnaire, CISS – Coping Inventory for Stressful Situations, WOC – Ways of Coping Questionnaire, FCSQ-14: Family Coping Strategies Questionnaire – 14 items, IBD-Cope: Inflammatory Bowel Disease Coping Inventory, JCS – Jalowiec Coping Scale, CORS: Children’s Outcome Rating Scale, SWLS: Satisfaction with Life Scale, SF-12: 12-Item Short Form Survey, BIPQ: Brief Illness Perception Questionnaire, BCOPR: Brief Coping with Problems Related to Health Questionnaire, PCS: Measures passive coping behaviors, IMPACT-III – Inflammatory Bowel Disease Questionnaire for Children, SF-36: 36-Item Short Form Survey, WPAI: Work Productivity and Activity Impairment Questionnaire, COPE Questionnaire: Coping Orientation to Problems Experienced Questionnaire, WHOQOL-Brief: World Health Organization Quality of Life - Brief Version

###  Theoretical Frameworks and Conceptualization of Coping Strategies in IBD

 Coping has been described in many articles, and there are currently more than 400 descriptions of people’s coping.^22^ It is broadly defined as individuals’ cognitive and behavioral efforts to manage internal or external demands exceeding their available resources.^23^ Later, coping is considered to be the healthy or unhealthy prevention, elimination, or attenuation behaviors that people take in response to stressors, intentionally or unintentionally, to minimize or eliminate distress.^24^ A coping behavior, intentional or unintentional, aims to prevent, alleviate, or eliminate distress caused by stressors.^8^ Although stressors, such as those associated with IBD, may persist, effective coping can mitigate emotional, social, and physical burdens.^25^ Researchers regularly divide these coping techniques into several groups, such as problem-focused as opposed to emotion-focused.^26^ The most representative and widely used ones are those from Lazarus and Folkman, who introduced the Stress-Coping Model in 1984. This model conceptualizes stressors as external pressures or emotional states perceived as challenging, while coping involves strategies adopted to mitigate these pressures.^27^

**Figure 2:**
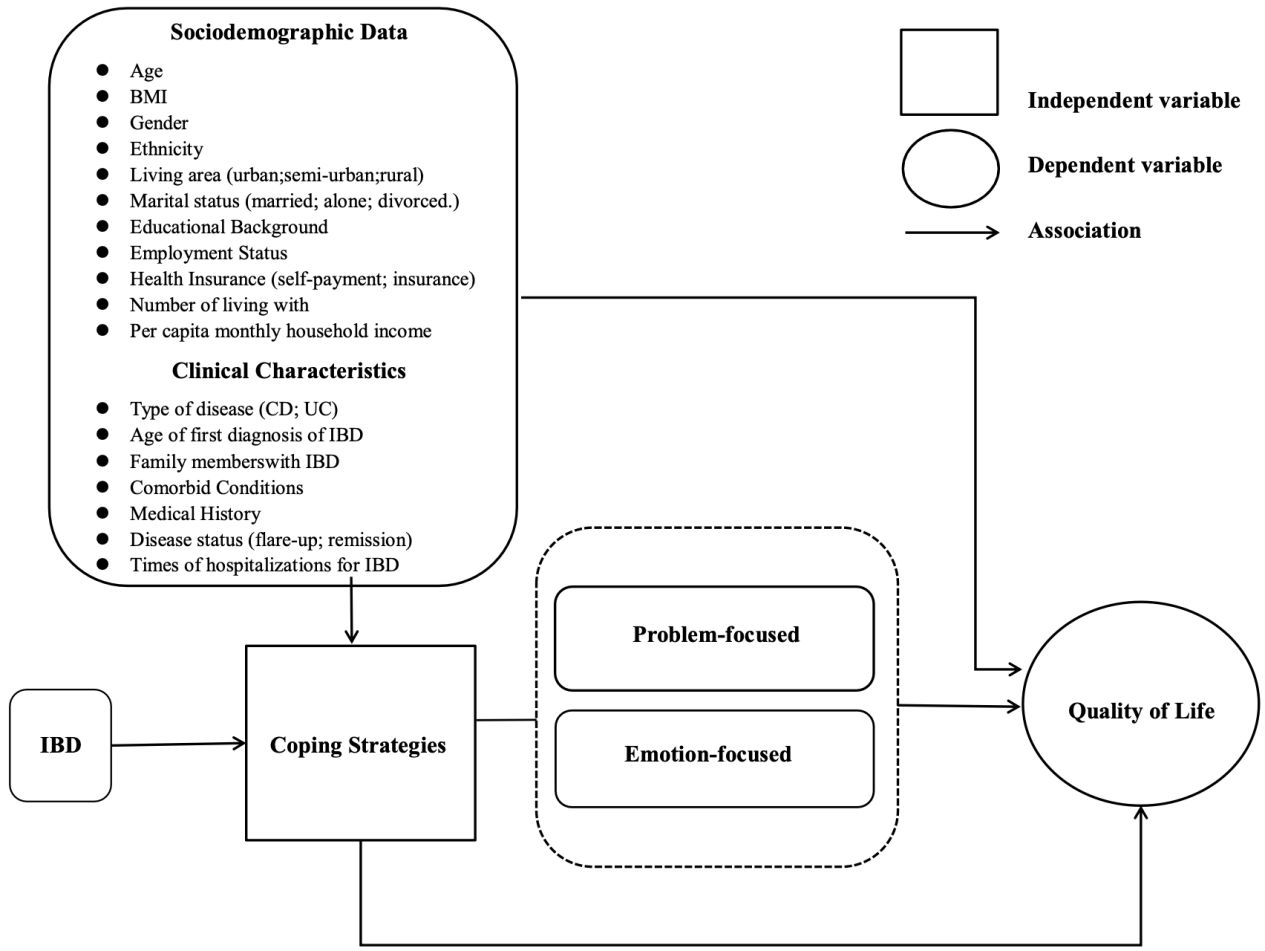
Conceptual framework of coping strategies and quality of life

 The Transactional Model of Stress and Coping (TMSC) builds upon the earlier framework, emphasizing the dynamic interaction between individuals and their environment.^28^ It focuses on how individuals evaluate stressors and select strategies to adapt to environmental changes. This model is particularly relevant in clinical contexts, providing insight into how patients with chronic conditions like IBD manage their symptoms and maintain well-being.^28^ This theoretical framework provides a foundation for understanding how coping strategies impact the QoL among patients with IBD by incorporating key sociodemographic and clinical factors. Figure 2 concisely represents this framework, highlighting the role of problem-focused and emotion-focused coping strategies in mediating the relationship between these factors and QoL outcomes. The model also underscores how independent variables, such as IBD characteristics and personal background, influence coping strategies, ultimately shaping the patient’s well-being.

###  The Relationship Between Coping and Quality of Life

 Given the unpredictable nature of IBD, various clinical and sociodemographic factors shape patients’ QoL. This review adopts a coping-centered perspective, revealing statistically significant associations between coping strategies and QoL across 17 studies. One study found that the way people deal with the problems caused by IBD has a significant impact on their QoL, and even after accounting for illness behavior, people who mainly used adaptive coping styles had significantly better QoL than those who mainly used maladaptive coping styles (*P* < 0.001).^11^ In addition, the study confirmed that confrontational, avoidant, and positive coping styles were used most often by people with IBD. Even when we considered how active the disease was, people who used adaptive coping styles had a better QoL than those who used maladaptive coping styles.^29^ Patients utilizing adaptive coping strategies, such as problem-solving or seeking social support, often exhibit better disease management and improved QoL, even when facing high disease activity levels.^11^ Teenagers with IBD were surveyed in China for a study that found a strong link between ways to cope and QoL. The research found that adaptive coping mechanisms were positively associated with QoL (β = 13.636, 95% CI: 3.090-24.182, *P* = 0.014), On the other hand, avoidance and negative emotional responses were indicative of maladaptive methods of coping that correlated to lower QoL (β = -13.636, 95% CI: -24.182 to -3.090, *P* = 0.014).^30^

 Coping not only directly affects patients’ QoL, but also works through indirect pathways. This indirect effect is particularly significant when psychological variables (such as coping style, mindfulness, and self-efficacy) partially mediate the relationship between illness cognition and psychological distress. Maladaptive coping, as a major factor, exacerbates psychological distress and has a greater negative impact on QoL.^14,16-18^ Although the measures of coping and QoL assessments varied among the included studies, there is ample evidence that coping plays an important role in QoL both directly and through indirect pathways.

###  Problem-Focused Coping 

 In the description of Lazarus and Folkman, problem orientation refers to individuals taking positive actions to solve or cope with specific stressful events or problems, that is, actively seeking solutions, making plans, and taking action. An investigation of illness-specific cognitive behavioral therapy (CBT) for anxiety and depression among IBD,^31^ revealed that patients with IBD who participated in CBT experienced significant improvements in mental health and QoL. Researchers believe CBT helps patients with IBD understand and learn positive thinking and effective coping strategies. Another study shows that when patients actively participate in formulation and planning, such as adopting positive problem-oriented behaviors such as facing the disease head-on, controlling diet, taking medications regularly, etc., it can lead to a better long-term prognosis and QoL.^32^ When this coping strategy is adopted, it can help individuals cope with practical problems and challenges in disease situations, enhancing the patient’s sense of control and ability to cope with the disease. However, some researchers have also pointed out that although problem-focused coping strategies may be beneficial, overreliance on these strategies to control IBD in the future may lead to poorer outcomes for patients psychologically due to stress.

###  Emotion-Focused Coping

 Emotion-oriented coping usually interacts with problem-oriented coping to jointly affect the individual’s response to stressful events and the results. The emotion-oriented approach aims to regulate an individual’s emotional response to reduce the negative emotional experience caused by stress. For example, patients express their illness experiences with peers or try some relaxation techniques to help themselves relieve stress,^33^ Research findings on emotion-oriented coping are mixed. In 2021, an online mindfulness intervention for patients with IBD showed that by organizing online mindfulness courses, participants’ measures of depression, stress, and anxiety were significantly reduced. However, there was no significant difference in disease status and life-related quality before and after.^34^ In addition, regarding patients with IBD, studies have consistently shown that emotion-focused coping strategies are associated with higher anxiety levels and lower QoL.^35-37^ Other than that, certain personality traits, alexithymia, basic perception, etc. are also related to negative coping outcomes.^38^ For example, a study indicates that patients with IBD with higher neuroticism scores are more likely to use negative coping strategies.^39^ Above all, emotion-focused coping may improve mental health or alleviate emotional distress but may not directly affect disease outcomes and may not effectively address the underlying problem.

## Discussion

 Compared to IBD management, research and application of coping strategies in other chronic conditions, such as diabetes and cardiovascular diseases, are more advanced. Theoretical frameworks in these fields have evolved from basic coping models to targeted interventions that are widely integrated into healthcare practices.^10,16,27^ This progression suggests that similar strategies could be adapted to IBD care to improve disease management and patient outcomes. Existing coping frameworks, while foundational for understanding coping behaviors, do not fully address the unique challenges faced by patients with IBD. IBD symptoms are often unpredictable, involving sudden physical discomfort and chronic psychological stress.^3^ To enhance these frameworks, it is crucial to incorporate IBD-specific issues, such as disease uncertainty, lack of social support, and diverse stressors.^19,28^

 Future research should develop tailored coping strategies that address the multidimensional needs of patients with IBD-spanning physical, psychological, and social domains. Additionally, targeted studies focusing on specific populations can explore the dynamic relationship between psychological factors and coping patterns, offering insights into the long-term effectiveness of different strategies.^14,16,29^ This will provide a scientific basis for optimizing personalized interventions that better align with the evolving needs of patients with IBD. The management of patients with IBD requires long-term support, which traditional care models may struggle to provide consistently. Integrating mobile applications, virtual tools, and digital platforms can enhance patients’ access to essential resources.^40^ For instance, smartphone-based apps that help patients monitor their condition, remind them to take medications, and offer psychological support can significantly improve self-management capabilities.^41^

 Additionally, combining virtual healthcare platforms with teleconsultation services ensures continuous professional support, minimizing disruptions caused by geographical or time constraints. Technological tools not only facilitate effective disease management but also enhance patient adherence. Research indicates that many patients prefer engaging with psychological interventions and support groups through virtual channels, which provide real-time feedback on their emotional well-being, allowing healthcare teams to promptly adjust interventions.^41,42^

 This review focuses exclusively on studies that use standardized tools to measure coping strategies in patients with IBD, which limits insights from qualitative research. This could lead to an incomplete understanding of patients’ subjective experiences and actual needs. Additionally, variations in measurement tools across countries may affect the comparability and consistency of results. Most included studies are cross-sectional or case-control designs, which are limited in their ability to establish long-term causal relationships between variables. Furthermore, since the studies encompass patients at different disease stages, coping strategies and their impact on QoL likely evolve as the disease progresses. This makes it challenging to pinpoint the specific effects of coping strategies at various stages of IBD. Future research should address these limitations by incorporating longitudinal designs and qualitative methods to capture the full spectrum of patient experiences.

## Conclusion

 The findings and limitations of this study highlight several gaps in research on the relationship between coping strategies and QoL in patients with IBD. Key shortcomings include a lack of studies focused on specific populations, insufficient multi-center research, limited longitudinal studies, cross-cultural inconsistencies in research tools, and inadequate attention to the evolving impact of coping strategies at different stages of the disease. These limitations restrict our understanding of the long-term effectiveness of coping strategies and their causal relationships with patient outcomes. Future research should prioritize multi-center studies using cross-sectional and longitudinal designs to capture more comprehensive data. Integrating qualitative research would also provide deeper insights into patients’ subjective experiences and individualized needs, which are often overlooked by quantitative methods. This approach can clarify the long-term effects of coping strategies and support the development of personalized interventions, advancing specialized IBD care. Moreover, future studies should expand the analysis within coping frameworks to identify predictive and risk factors affecting both coping and QoL. This would enable clinicians to implement early interventions, ensuring timely and effective care for patients with IBD as their disease progresses.
